# Comparative evaluation between glass and polyethylene fiber reinforced composites: A review of the current literature

**DOI:** 10.4317/jced.54205

**Published:** 2017-12-01

**Authors:** Enas Mangoush, Eija Säilynoja, Roosa Prinssi, Lippo Lassila, Pekka K. Vallittu, Sufyan Garoushi

**Affiliations:** 1 Turku Clinical Biomaterial Center -TCBC, Institute of Dentistry, University of Turku, Turku, Finland; 2 Reseach Development and Production Department, Stick Tech Ltd – Member of GC Group, Turku, Finland; 3 Department of Biomaterials Science, Institute of Dentistry, University of Turku; 4 City of Turku Welfare Division, Oral Health Care, Turku, Finland

## Abstract

**Background:**

Fiber reinforced composite (FRC) is a promising class of material that gives clinicians alternative treatment options. There are many FRC products available in the market based on either glass or polyethylene fiber type. The aim of this study was to present a comparison between glass and polyethylene fiber reinforced composites based on available literature review.

**Material and Methods:**

A thorough literature search, with no limitation, was done up to June 2017. The range of relevant publications was surveyed using PubMed and Google Scholar. From the search results, articles related to our search terms were only considered. An assessment of these articles was done by two individuals in order to include only articles directly compare between glass and polyethylene FRCs. The search terms used were “fiber reinforced dental composites” and “glass and polyethylene fibers in dentistry”.

**Results:**

The search provided 276 titles. Full-text analysis was performed for 29 articles that met the inclusion criteria. Most were laboratory-based research with various test specimen designs prepared according to ISO standard or with extracted teeth and only three articles were clinical studies. Most of studies (n=23) found superior characteristics of glass FRCs over polyethylene FRCs.

**Conclusions:**

Significant reinforcement differences between commercial glass and polyethylene fiber reinforced composites were found.

** Key words:**Fiber reinforced composite, glass fiber, polyethylene fiber.

## Introduction

Dentistry has rapidly developed during the last few decades, where variety innovative techniques have changed the conventional treatment methods as applications of new dental materials give better outcomes than traditional ones. Resin-based dental composite is one of those materials which undergoes through many changes and improvements (1). One of the most effective changes was the incorporation of fibers with particulate filler composite resin (PFC). Generally, mechanical properties of fiber reinforced composites (FRCs) structures have been found to be superior to that of non-reinforced composites *in vitro* (2). FRCs technology may solve many of the problems associated with a metal alloy substructure. When compared with metal alloys, FRCs offer many other advantages including non-corrosiveness, translucency, lower cost, higher aesthetic, good bonding properties and repair facility (3). Furthermore, their strength to weight ratios are superior to those of most alloys. In addition to mentioned merits, FRCs give alternatives for both direct and laboratory fabrication (3).

The typical FRC materials are made of polymer matrix that is reinforced by ﬁne fibers. The FRC structure consists of light cure monomers, having the function of holding the fibers together in the composite structure, whereas fiber is the reinforcing part providing stability and stiffness (1). FRCs are currently commonly used in several fields of dentistry such as fixed prosthodontics, restorative dentistry, periodontology, and in repairs of removable prosthodontic devices (4). The common types of fibers used in dentistry are glass and polyethylene fibers. Glass fibers have high tensile strength combined with low extensibility. Their transparent appearance makes them well suited for dental applications with high cosmetic demands (4). The components of glass fibers can be classified into six categories depending on their composition and application (A-, C-, D-, E-, R- and S-glass) with difference in mechanical and chemical properties (4). Polyethylene fibers are one of the most durable reinforcing fibers available. They are made of aligned polymer chains, having low modulus and density, and presents good impact resistance. They are white in colour and thus it is possible to use them in aesthetic dental applications (5).

The effectiveness of fiber reinforcement technology is dependent on many variables including the type of resin used, the quantity of fibers in the resin matrix, fiber type, length, form, orientation, adhesion to the polymer matrix and impregnation of fibers with the resin (4,5). The use of each type of fiber within FRC structure has its own properties and advantages over the other type, therefore awareness of the advantages and limitations of each type of fiber will enable the clinician to select the best FRC for a particular clinical situation. There is limited number of literature founded that compare between the two FRCs (glass and polyethylene fibers based), and to make it easy for the dental practitioners to find out the appropriate material to use in dental office, the aim of this review is to make a general comparison in various properties of the glass and polyethylene fiber reinforced composites based on published scientific papers and available literature review.

## Material and Methods

Eligibility Criteria: Articles eligible for inclusion in this review were published in English language, peer-reviewed dated from 2002 to 2016. The articles selected had to include the search terms either in the title or abstract. Published articles and literature reviews that were full text were preferred. Articles that were unpublished, personal communications, background information, and advertisements were filtered and excluded.

Data Sources: A literature electronic search was performed through PubMed and Google Scholar. The search terms used were, fiber reinforced dental composites, and glass and polyethylene fibers in dentistry.

**Figure 1:**
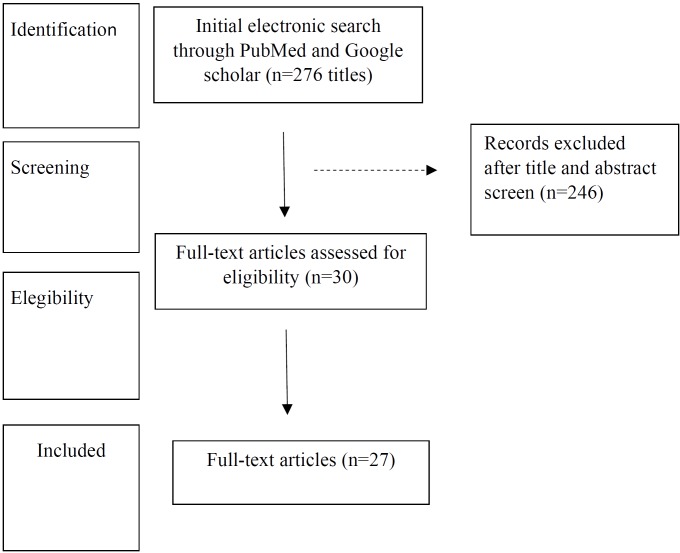
Flow diagram (PRISMA format) of the screening and selection process.

Search Strategy: After omitting the duplicates/repetitive articles, a total of 29 full text articles were studied and concluded for review (Fig. 1). Two investigators independently searched and screened the results using the agreed inclusion criteria.

• Laboratory or clinical study

• Direct compare between dental glass and polyethylene FRCs

• Published in peer-reviewed journal

• Reported details of manufacturer’s information

• Language: English

Studies not meeting all inclusion criteria were excluded from the review. Moreover, publications were excluded if they were only cases reports.

Data Extraction: Only articles considered relevant to the objectives of this review were considered. Articles included that directly compare between commercial glass and polyethylene fiber reinforced composites in different characteristics and properties.

Commercial fiber reinforced composites (FRCs) used in the reviewed studies are listed in  Table 1.

**Table 1:**
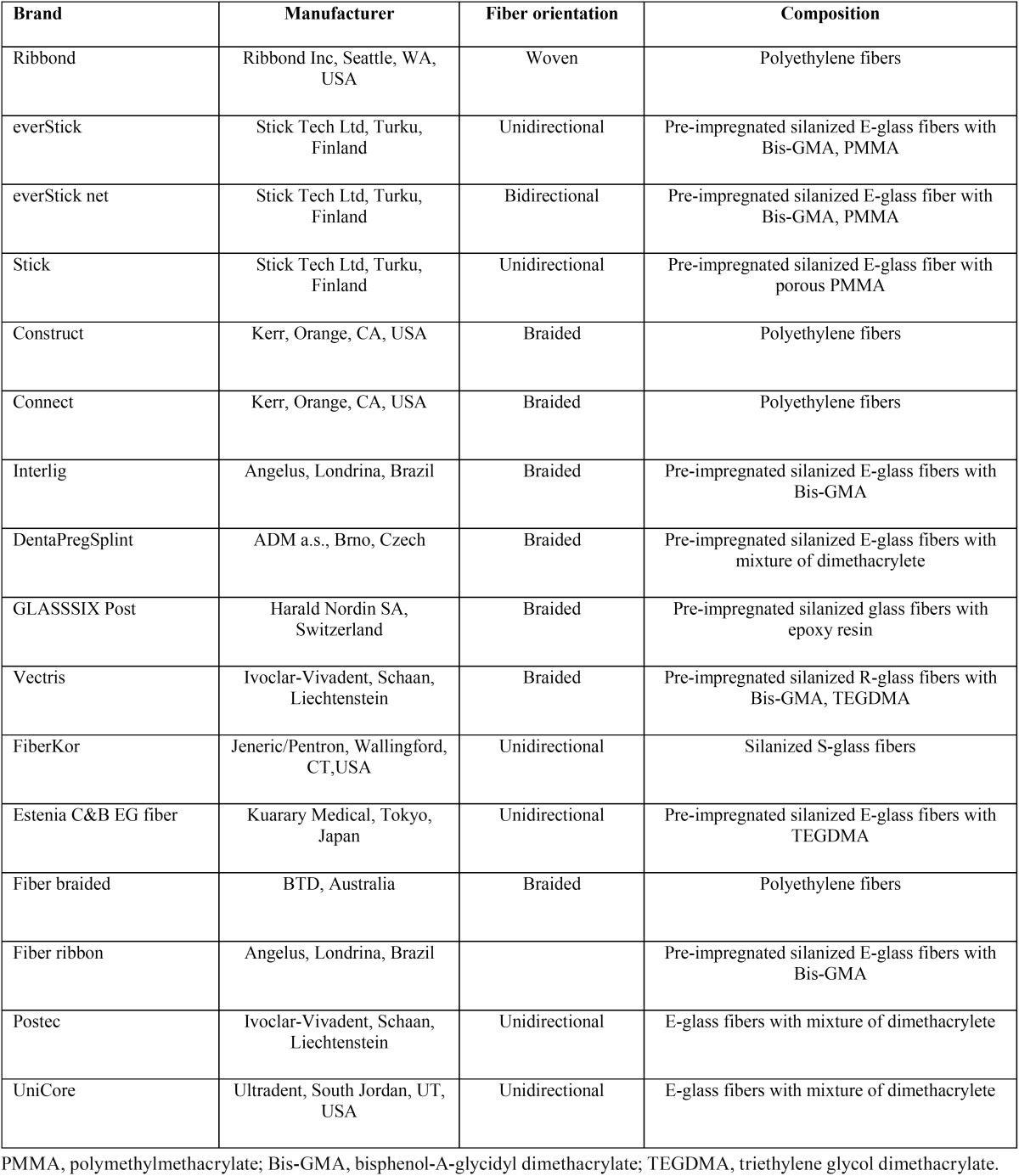
The FRCs investigated and their composition.

## Results and Discussion

A thorough electronic literature search was done, using PubMed and Google scholar. After exclusion of repetition a number of 29 full text published articles was included, only articles which contain direct comparison were selected, 3 articles were clinical studies and 26 *in vitro* studies ( Table 2). Depending on available related data on those papers, material characteristics are compared, the majority of papers (79.3%) showed superior results of glass FRCs over polyethylene FRCs, 13.8% showed no differences between the two materials, and only 6.9% showed that polyethylene FRCs have better characteristics than glass FRCs. After detailed and thorough study of the whole reviewed articles, tested characteristics are divided into 4 main headlines, each headline contains one or more properties to be compared.

**Table 2:**
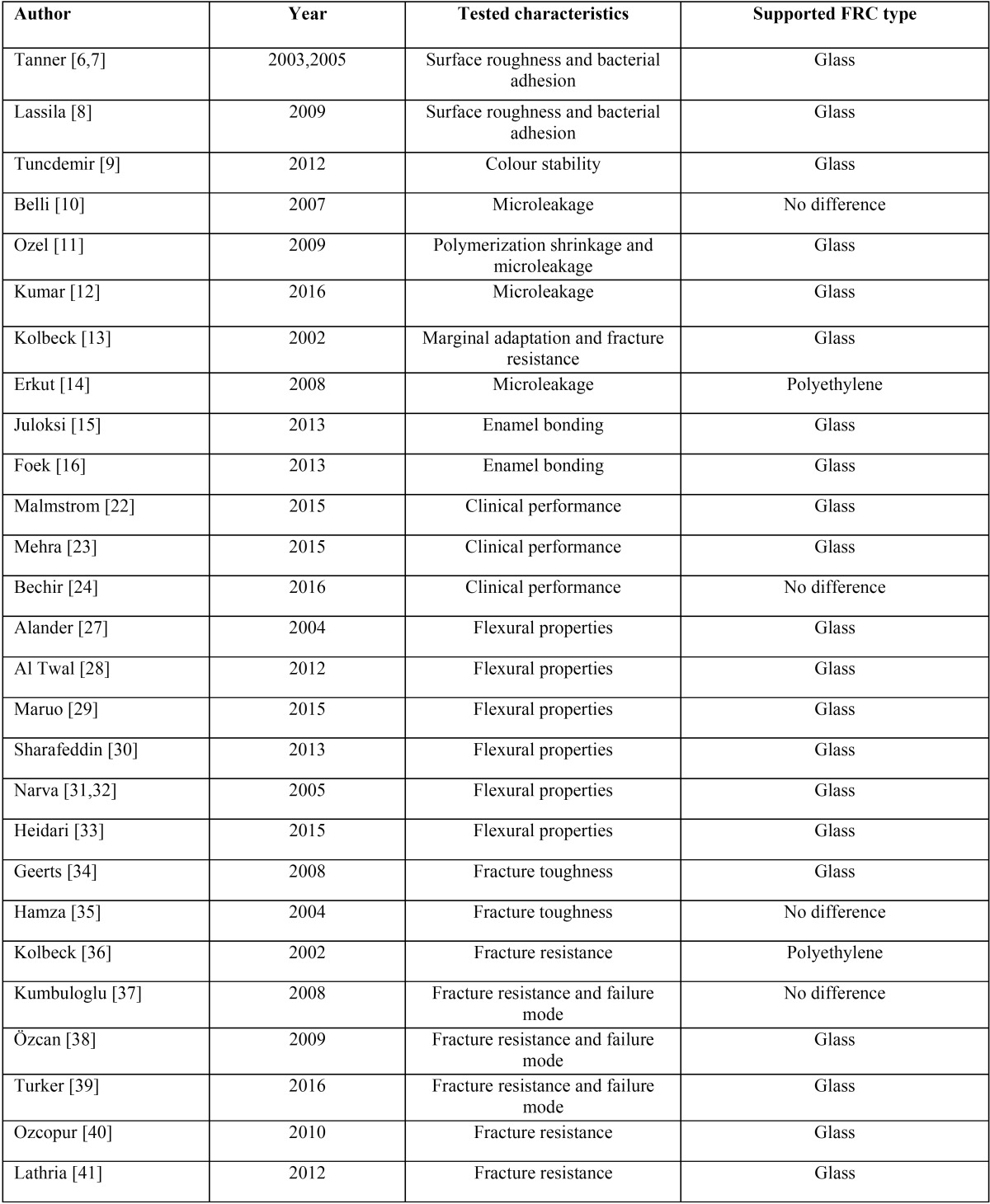
Details of studies included in the review.

1. Surface roughness and colour stability 

The rough surface of a restoration increases plaque accumulation, which may result in discoloration and increased the risk of secondary caries. Roughness can be related to a combination of factors that include the characteristics of the matrix, ratio and size of enclosed fibers, exposition of these fibers and formation of air bubbles during material preparation (6). Tanner *et al.*, studied the early plaque accumulation and bacterial adhesion (*Streptococcus mutans*) of two different experimental FRC materials (glass and polyethylene) in comparison with other commercial restorative materials (6,7). They reported that, in the absence of a salivary pellicle, polyethylene FRC showed the highest binding of *S. mutans*, and its surface was found to be much rougher than other materials tested (6). In the oral environment (*in vivo*), polyethylene FRC also displayed the highest counts of plaque accumulation and adhesion of *S. mutans* than glass FRC and other tested restorative materials (7). On other hand, glass FRC and particulate filler composite (PFC) showed similar plaque accumulation properties. Authors attributed this to similarity in their surface physico-chemical properties. They are both composites composed of inorganic filler components in an organic polymer matrix (7). These finding are supported by Lassila *et al.*, who studied the surface roughness and bacterial adhesion of FRCs and conventional restorative materials (8). They showed that polyethylene FRC (Ribbond) specimens had the highest surface roughness value, which is calculated around 2.33 μm, while that of glass FRC (everStick) is almost half the value of polyethylene FRC (8).

Discoloration of tooth-coloured composite materials is one of the most common reasons for the replacement of prosthetic facing and restorations in aesthetic areas. After insertion of reinforcing fibers as an intermediate layer in composite resin to reinforce the composite, some compositional changes happened in different ways depending on fiber type.

Tuncdemir *et al.*, had studied the effect of FRCs on the colour changes and stability of resin composites before and after accelerated aging (9). They used two types of FRCs, polyethylene (Ribbond) and glass FRCs (everStick net) in addition to plain anterior and posterior PFCs. Before accelerated aging, they found that the types of PFC and FRC materials used accounted for the colour difference. Anterior PFC reinforced by Polyethylene FRC resulted in a larger total colour change (ΔE=1.00) than with glass FRC (ΔE=0.32). After 300 hour of accelerated aging (which is equivalent to 1 year of clinical service) the FRCs showed clinically noticeable colour changes in the range of 1.5–3 NBS units. Therefore, reinforcement of particulate composite with FRC would not improve the colour stability of composite resins subjected to accelerated aging. According to CIE colour tolerance specification, colour changes of 1–2 ΔE units can be perceived by the human eye and those lower than 3.3 ΔE units are clinically acceptable (9). Therefore, the FRCs materials demonstrated clinically acceptable colour change after aging (ΔE<3.3). They also founded that posterior composite reinforced with polyethylene FRC exhibited the largest colour change while the anterior composite reinforced with glass FRC showed the lowest colour change. These colour change results could be linked to their chemical structures (9). Glass is an amorphous material consisting of silica tetrahedral bonded together in a random network. This structure is different from that of organic fibers such as polyethylene fibers (9). Materials with inorganic content reportedly had a lower water sorption rate and lower discoloration incidence than that of organic content. Thus, differences in their chemical structures could be the reason of why polyethylene FRCs exhibited greater colour change than glass FRCs (9).

2. Polymerization shrinkage and microleakage

Polymerization shrinkage is one of the most critical limitation of the light cured dental composites. Such shrinkage induces contraction stress at interface between composite resin and cavity walls leading to gap-formation and secondary caries. This is a major problem in current restorative dentistry. Many studies have reported efforts to develop methods to eliminate this problem. One of these recommended method is by placing a layer of FRC base in order to reduce the polymerization shrinkage stress and microleakage.

Belli *et al.*, evaluated the effect of different FRCs (polyethylene and glass) on microleakage of class ІІ composite restorations (10). Their results indicated that there was no significant difference in microleakage among the groups when the cavities were lined with glass FRC (everStick net) or polyethylene FRC (Ribbond). Authors stated in conclusion that both polyethylene and glass FRCs in combination with flowable composite helps to reduce occlusal leakage in Class II adhesive cavities with enamel margins (10).

Ozel and Soyman evaluated the effects of fiber net on polymerization shrinkage and microleakage of MOD composite restorations (11). Two types of FRC nets, polyethylene (Ribbond) and glass (everStick net) were placed as base on gingival and axial cavity walls. Their results revealed that composite restorations reinforced by glass FRC when compared with polyethylene FRC, showed less microleakage scores, but the difference was not statistically significant (11). Additionally, volumetric polymerization shrinkage of composite resin reinforced by glass FRCs was 1.87 % ± 0.04 which was lower than polyethylene FRC reinforced group 1.95 % ± 0.06. Authors attributed this difference to the difficulty in obtaining good adhesion between the polyethylene fibers and resin matrix (11). This is in accordance with Kumar *et al.*, who demonstrated that microleakage score (MS) at gingival margin of class II composite restoration reinforced by glass FRC (MS 3.2, everStick net) was statistically lower than polyethylene FRC (MS 4.2, Ribbond) restorations (12).

To compare the marginal adaptation of both polyethylene and glass FRCs prosthesis, Kolbeck *et al.*, carried out *in vitro* study with a total of 16 three-unit fixed partial dentures (FPDs) of each material combination were manufactured and adhesively luted to human molars (13). Before and after thermo-cycling, the quality of marginal adaptation was examined by two different tests (SEM and dye penetration). The marginal quality of FPDs, showed statistically significant better results for the glass FRC (FiberKor) restorations ‘with 98% of perfect marginal areas’ than for the polyethylene FRC (Connect) ‘with 82% perfect marginal areas’ before as well as after aging process (13).

Erkut *et al.*, evaluated microleakage in over flared root canal teeth restored with different types of FRC posts (14). Individually shaped polyethylene (Ribbond) and glass (everStick) FRC posts were used with another two prefabricated types of FRC posts. The resulted SEM photomicrographs data clarified that individually shaped polyethylene and glass FRC groups showed less detachment and better continuity at the luting-resin-dentin interface compared with prefabricated FRC posts. However, among the individually shaped posts, polyethylene reinforced posts showed a lower microleakage at the coronal section compared with the other groups (14). Although both individually made FRC posts were formed according to post space, in the glass FRC post group, the polymerized post was removed from the canal and luted into the post space with a resin based luting material. While in the polyethylene post group, the FRC and the luting resin polymerized together in situ. This actually explains the results since resin shrinkage is minimal and is restricted by distribution of the fibers.

3. Bonding and clinical performance

The performance of the FRC system depends on the cohesive strength of the polymer matrix as well as fiber type, volume fraction, and the quality of the fiber-polymer matrix interface. In addition to the mechanical performance, the composition of the polymer matrix and fibers also has a major role in the bonding ability and durability of FRC to the luting resin cement and the tooth-restoration interface (3-5). Juloski *et al.*, studied the effect of glass and polyethylene FRCs insertion on shear bond strength of unground human enamel (ESBS) (15). They used different products of polyethylene FRC (Ribbond, Connect and Construct) and glass FRC (everStick). It was resulted that statistical significant differences in ESBS were founded among the groups. According to post-hoc test, glass FRC yielded a significantly higher ESBS (23.24 ± 5.81 MPa) than the polyethylene groups (18.59 ± 5.67). This difference can be attributed to the different polymer matrix composition and method of impregnation with resin and fiber orientation (15). Glass FRC (everStick) is pre-impregnated with the light-polymerizable dimethacrylate resin system containing linear polymer phases, which forms semi-IPN polymer network after being polymerized offers better bonding site for composite resin and tooth structure (4).

In another study by Foek *et al.*, debonding force and failure type of different FRC lingual retainers were analysed after 10,00,000 fatigue loading cycles (16). They used different products of glass FRC (everStick, Interlig and DentaPregSplint) and polyethylene FRC (Ribbond).

Fatigue created by cycling loading did not cause failure of the FRC retainers. Regarding debonding force, glass FRC (Denta-PregSplint) had higher value (830 ± 258 N) than polyethylene FRC (731 ± 329 N), although the difference was not significant (*p*>0.05).

Authors emphasised that failure types deserve more attention than the debonding performance of the tested FRC materials. The failure types should be evaluated with regard to not only the adhesion quality but also clinical reversibility of treatment (16). The failure type is varied between the glass and polyethylene FRCs, 80% of failures of the glass FRC (everStick) presented partial adhesive debonding of the retainer from one of the teeth, while 20% presented complete adhesive debonding. On the other hand, failure type of polyethylene FRC showed complete adhesive debonding and material fracture in 50% and 40% of the specimens, respectively (16). Authors pointed out that resin adhesion to polyethylene FRC might be less favourable mainly because of the difficulty in plasma coating, salinization and impregnation of polyethylene fibers (16,17). The quality of the interface between the reinforcing fibers and the resin matrix affects the performance of the FRC materials. Without adequate adhesion between these phases, the fibers act as voids in the resin matrix, thereby weakening the FRC structure (18,19). For glass FRC materials, adhesion was promoted with the use of silane coupling agents, which are known to maximize chemical and physical bonding between the different components within composite materials (20). It was shown with scanning electron microscope and nanoindentation studies that silanization of glass fibers enhanced the adhesion between the fibers and the organic resin matrix (18,20). Although, silanation processes and silane compounds may vary between glass FRC products and that directly effect on the hydrolytic stability of the material. Interestingly, Vallittu studied the effect of 10 years of water immersion on the flexural properties of FRC materials. He showed that reduction in flexural strength and modulus of E-glass FRC were 24% and 21%, respectively, and for silica FRC reductions were 47% and 46%, respectively (21).

Resin bonded FPD is a valid treatment option for the replacement of missing teeth in circumstances when the conservation of the tooth structure is needed. To assess its success and clinical performance, a two-year clinical study was done by Malmstrom and his colleagues (22). In their study 167 FRC FPDs were directly fabricated to restore a single missing tooth (anterior or posterior). The recipients were randomised into two groups according to FRC materials used (everStick or Ribbond). After two years follow-up period, there were no statistically significant differences in survival rate between groups with different fiber materials (*p*>0.05). However, among 17 failed FRC FPDs, 65% were made of polyethylene FRC and 35% were made of glass FRC. In terms of failure distribution, it was founded that in one end debond failure type, all the failed prosthesis are made from polyethylene FRC. And in case of fracture/delamination of veneered composite failure type, 56% of failures resulted with the usage of polyethylene FRC while 44% of failures following the use of glass FRC (22).

The use of both glass and polyethylene FRC posts to restore grossly destroyed primary incisors is a challengeable treatment option. Mehra *et al.*, reported one year data of 45 grossly decayed primary anterior teeth, which were endodontically treated and fiber posts (glass and polyethylene) placed in the canals (23). After an interval of 12 months, significant difference (*p*<0.05) was observed between different FRC posts. 86.7% of the teeth exhibited complete retention of the polyethylene FRC posts (Ribbond), and 93.3% of the teeth exhibited complete retention of the glass FRC posts (GLASSSIX). Moreover, marginal discoloration comparison was done after 12 months, and 20% of the teeth exhibited marginal discoloration in polyethylene FRC post group, while only 13.3% in glass FRC post group. Authors stated that durability of glass FRC posts may be attributed to the individual fiber quantity, composition of resin matrix (epoxy resin with approximately 65 vol% glass fiber content) and the adequate adhesion of the fibers to the polymer matrix (23).

Because of brittleness and natural rigidity of conventional composite resins, the composites as splints with metal wires are prone to failure. To overcome this clinical limitation, FRC has allowed clinicians to replace metal wires. Different types of commercial FRC splints are available for the purpose of conservative splinting. Bechir *et al.*, evaluated some aspects in the clinical effectiveness of FRCs periodontal splints on mobile anterior teeth (grade I & II) of 62 patients (24). Two types of FRC splints were used, glass FRC (Interlig) and polyethylene FRC (Ribbond). After 6 months of periodic follow up monitoring, it was founded that there is no significant difference in the degree of separation/adhesion failure or in the patient’s comfort. Both types of FRCs splints had beneficial effects in decreasing the degree of dental mobility and it was remarked their aesthetic acceptability (24). Nevertheless, more reduction of tooth mobility appeared in polyethylene FRC group (96.04%), compared to glass FRC group (95.80%), and duo to the lack of significant differences detected between the two fibers in the mobility degree, its considered that both types of FRC splints have demonstrated their excellent aesthetic acceptability and comfort (24).

4. Mechanical properties and load bearing capacity 

Many studies have approached to find a way to improve the mechanical properties of PFCs (1). These include choosing a suitable resin matrix, using different curing methods, and improving the filler content. Nonetheless, these substances do not have the adequate flexural strength to replace the lost tooth structure (1,25). Combination of PFC with FRC has already shown an improvement in mechanical properties when used *in vivo* (25). The fracture related material properties, such as fracture resistance, deformation under occlusal load, and the marginal degradation of materials have usually been evaluated by the determination of the basic material parameters of fracture toughness and flexural strength and modulus (26).

Alander *et al.*, had determined the flexural stress at the initial and final failure points of six commercially available FRCs (glass and polyethylene) used in the framework construction of FPDs (27). They found that the highest measured stress required for initial failure (579 MPa) and the ultimate flexural strength (764 MPa) were obtained with glass FRC (everStick). While the lowest values of initial failure stress (107 MPa) and ultimate flexural strength (132 MPa) were recorded with polyethylene FRC (Rib-bond). The initial stress required for starting breakage was three times higher in glass than polyethylene FRCs. The authors explained that the possible cause of variations may be attributed to the existence of voids in the manually impregnated FRCs (polyethylene) and also weakening of the fiber-polymer matrix interphase (27). This in line with Al Twal *et al.*, who determined the effect of glass (everStick) and polyethylene (Ribbond) fibers reinforcement on the flexural property of two temporary composite resins (28). In their study, reinforcement with glass fibers significantly (*p*<0.05) increased the mean flexural strength and flexural fatigue limit of both composite resins used. The strengthening effects of glass FRC was more than double of polyethylene FRC. The mean flexural strength values of the two types of composite resin with glass FRC was 274.3 and 438.1 (MPa), while with polyethylene FRC was 132 and 130 (MPa). Authors claimed that differences in results are due to differences in fiber architecture and orientation, and also to the better bonding of pre-impregnated glass fibers to the composite resin (28).

In addition to the above mentioned studies, Maruo *et al.*, and Sharafeddin *et al.*, used also polyethylene and glass fibers to reinforce different PFC for prosthetic frameworks. Maruo *et al.*, reported that in contrast to glass fibers reinforcement, addition of polyethylene fibers in woven form (Construct) did not increase or improve the flexural properties of composite resins, except for improving the specimens displacement (29). Authors explained the discrepancy between their study and previous studies stemmed from a difference in the degree of fiber distribution in the resin matrix (29).

Sharafeddin *et al.*, stated that, the reasons that the applied glass FRC (Fiber ribbon) had more strength than polyethylene FRC (Fiber braided) was its pre-impregnation when manufactured (30). They claimed that Pre-impregnation improves the bonding properties of the fibers and creates a homogenous substance, which in turn, increases the strength 2 to 3 times more than manually impregnated fibers (30).

In further corresponding studies, Narva *et al.*, determined the static and fatigue flexural properties of denture base polymer (PMMA) that had been reinforced with glass and polyethylene FRCs (31,32). The results of these studies showed clear statistically differences between glass and polyethylene FRCs in denture base reinforcement. Flexural strength and modulus value of denture base specimens reinforced by glass FRC (everStick) were 266 MPa and 5.3 GPa respectively, while that of polyethylene FRC (Ribbond) were 151 MPa and 3.5 GPa respectively (31). Interestingly, denture base specimens reinforced by glass FRC were most effectively hindered the crack propagation during dynamic loading in comparison to polyethylene FRC reinforced specimens (32). When testing polyethylene FRC (Ribbond) specimens, the number of cycles to failure and mean force required to cause 1 mm deflection was 23 220 cycles and 41.4 N, respectively. While glass FRC (Stick) specimens maintained their fatigue resistance at the maximum of 100 000 cycles and mean force to cause deflection was 61.9 N. The variations in the result attributed to two factors; fiber orientation and interfacial adhesion between FRCs and highly viscous denture base resin. According to fiber geometries and Krenchel’s factor, unidirectional reinforcement has the most efficient reinforcing factor than any other form of reinforcement, which can be one reason for the lower flexural strength in specimens reinforced by polyethylene FRC (4,25). It is also possible that impregnation of polyethylene fibers by the highly viscous denture base resin was inadequate (31,32). This is in accordance with Heidari *et al.*, who evaluated the flexural strength of glass and polyethylene FRCs combined with cold and heat cure denture base materials (33). They reported that flexural strength created with glass FRCs was greater than that of polyethylene FRCs (33).

The fracture toughness of a material is a measure of how well that material hinders the progress of a crack or flaw under load. Fracture toughness correlates to the fracture energy that is consumed in plastic deformation and proposes to approximate the crack growth rate (26). During mastication, the ability of a restorative material to withstand fracture critically depends on the growth of micro- and macro-voids, and mechanisms of propagation of micro-cracks (26).

Geerts *et al.*, studied the effect of glass and polyethylene fibers reinforcement on the fracture toughness of two types of materials (PMMA and PFC) used for provisional restorations (34). As the previous studies, it was resulted that glass fibers (everStick) reinforcements produced significantly higher fracture toughness for both PMMA and PFC (40.0 and 46.7 MNm-1.5) respectively. But the polyethylene fibers (Construct) did not (29.7 and 35.7 MNm-1.5) in comparison with unreinforced materials (34). Geerts *et al.*, concluded that, wherever aesthetic and space are of concern, glass fiber reinforcement seems to be the most appropriate of the methods tested for reinforcing both PMMA and PFC resins. On contrary, Hamza *et al.*, reported no statistical significant differences between glass and polyethylene fibers reinforcement on the fracture toughness and flexural strength of different provisional resin materials (35).

To compare the load bearing capacity of both polyethylene and glass FRCs prosthesis, Kolbeck *et al.*, carried out *in vitro* study with a total of 16 three-unit FPDs of each material combination were manufactured and adhesively luted to human molars before mechanical loading (13). The analysis of fracture resistance showed that, the median fracture strength of the polyethylene FRC (Connect) was 830 N, compared to 884 N for glass FRC (FiberKor), which is statistically not significant. Authors explained their results by the higher strength given by unidirectional orientation of glass fibers in comparison with woven polyethylene fibers reinforcement (12). Woven orientation cause a higher degree of flexibility of polyethylene FRC framework and thus an earlier shearing off of the veneering composite which cannot withstand the high bending tendency of the framework. In addition, the pre-impregnation of the glass FRC gives more reinforcing effect than plain polyethylene FRC, which is explainable with two reasons. First, a higher compound of framework and veneering material can be reached with pre-impregnated fibers, because the prefabricated wetting is better than the wetting of the fibers by hand. Secondly, there is a chemical bond between glass-fibers and veneering but not between polyethylene-fibers and veneering (13).

Interestingly, another study by the same authors (Kolbeck *et al.*) and materials, polyethylene fiber reinforced inlay-FPDs had statistically higher load bearing capacity than glass fiber reinforced inlay-FPDs (36). Authors claimed that higher fracture resistance of the polyethylene fiber constructions in this study might be explained by the fact that inlay FPDs are smaller than conventional FPDs and thus must withstand lower bending forces (36).

In another *in vitro* study, Kumbuloglu *et al.*, compared the fracture strength and failure mode of direct, surface retained, anterior FPD reinforced with either polyethylene or glass FRCs (37). Authors reported no significant differences were found among the polyethylene and glass FRCs restorations. Also, the failure type was almost the same for the two types of FRCs, with the most failure type as a detachment or delamination of veneering composite from the FRC framework, followed by chipping in the veneering composite (37).

FRC endodontic posts have been introduced to be used instead of metal alloys and ceramics (3). Their main proposed advantage was that they were more flexible than metal posts and had approximately modulus of elasticity (stiffness) as dentine. When bonded in place with resin cement, it was thought that forces would be distributed more evenly in the root, resulting in fewer root fractures (3,4). However, the success of restoring endodontically treated teeth (ETT) with FRC posts are strongly dependent on several microstructural parameters, to assess that, Özcan and Valandro compared the fracture strength and failure mode of different FRC posts (glass and polyethylene) and composite cores in teeth without coronal tooth structure (38). It was founded that the fracture load of composite core reinforced by glass FRC post (everStick) was 321 N and in groups reinforced by polyethylene FRC post (Ribbond) was 267 N. When differences in failure type was assessed, they found that glass FRC post remained intact in the root canal, and the core material was partially detached from the post. On the other hand, in polyethylene FRC post the failure was exclusively loss of post-core retention at post-hole opening. This leads to the result that polyethylene FRC posts were not able to support the covering resin core material (38). Authors concluded that the use of polyethylene FRC post was less favourable than glass FRC post. In the same way Turker *et al.*, determined the fracture resistance and the mode of fracture of endodontically treated canines restored with different FRC posts (39). The analysis of fracture resistance showed that, the mean fracture load value of the polyethylene FRC post (Connect) was 316 N, compared to 396 N for glass FRC post (Postec), which was not statistically significant. From other important point of view, the highest post dislodgment failure result (80%) was obtained from the polyethylene FRC post specimens, which also showed higher tendency of root fracture failures (39).

Confirming the previous mentioned studies, Ozcopur *et al.*, tested the effect of different FRC post systems (glass and polyethylene) on fracture strength of teeth with either sound root or with re-attached fractured fragments (40). Fracture strength among the roots with re-attached fragments revealed no significant differences. On the other hand, sound roots that were restored with glass FRC (Unicore) post displayed higher fracture strength values (1473 N) than polyethylene FRC (Ribbond) post (977 N).

In another congruous study, Luthria *et al.*, also evaluated the fracture resistance of ETT with wide MOD cavities restored with PFC resin reinforced by different types of FRCs (glass and polyethylene) (41). The results revealed that, the fracture resistance of composite restorations reinforced with glass FRCs (Interlig) was higher (600.5 N) than polyethylene FRCs (Ribbond) (514.6 N). Authors attributed the lower reinforcing efficiency of polyethylene FRC to the manually, non-uniform wetting of polyethylene FRC with unfilled resin, which negatively influenced on the adhesion of FRC to PFC resin matrix (41).

## Conclusions

Based on the above mentioned literature, polyethylene and glass FRCs have been used to reinforce resin matrix and their strength and reinforcement are among the highest to be found. Polyethylene FRCs are known for their biocompatibility and can be surface treated by irradiation (electrical plasma-treatment) to enhance bonding to resin. Despite the plasma treatment, the low surface energy and poor adhesion associated with polyethylene fibers makes the bonding between resin matrix and fiber not sufficient as it is. Glass FRCs are the most widely used reinforcing fibers with high tensile strength. Several authors have reported that glass FRCs have excellent mechanical properties with a relatively high modulus of elasticity and good bonding strength to the resin matrix. For dental applications, it’s recommended that FRCs should be impregnated with resin made by manufacturer, in order to ensure complete impregnation, thereby allowing the resin to come into contact with every fiber. There is wide variation between products in respect of fiber surface treatments and methods of incorporating the fibers into the resin matrix. Pre-impregnated glass FRC with light curing monomers which cross-link during polymerization of the overlying composite, forming a multiphase polymer network. This offers advantages of handling properties and bonding of fiber reinforced structure with composite resin (luting cement, veneering composite). Good adhesion between FRC and composite resin is one of the most important factor in reinforcement and load transfer from the surface to the fibrous structure and tooth.

Finally, it must be emphasized to dental clinicians that FRC products offered many alternative treatment options to the profession, but we should emphasis the correct selection and use of the material in order to achieve the reinforcement benefits and durability.
